# Association between vitamin D deficiency and allergic symptom in pregnant women

**DOI:** 10.1371/journal.pone.0214797

**Published:** 2019-04-10

**Authors:** Kumiko T. Kanatani, Yuichi Adachi, Kei Hamazaki, Kazunari Onishi, Tohshin Go, Kyoko Hirabayashi, Motonobu Watanabe, Keiko Sato, Youichi Kurozawa, Hidekuni Inadera, Hiroshi Oyama, Takeo Nakayama, for the Japan Environment and Children’s Study Group

**Affiliations:** 1 Department of Health Informatics, Kyoto University School of Public Health, Kyoto, Japan; 2 Kyoto Unit Centre for Japan Environment and Children’s Study, Graduate School of Medicine, Kyoto University, Kyoto, Japan; 3 Department of Pediatrics, Faculty of Medicine, University of Toyama, Toyama, Japan; 4 Department of Public Health, University of Toyama, Toyama, Japan; 5 Department of Public Health, Tottori University, Yonago, Tottori, Japan; 6 Doshisha University Center for Baby Science, Kizugawa, Kyoto, Japan; 7 Institute for Advancement of Clinical and Translational Science, Kyoto University Hospital, Kyoto, Japan; 8 Department of Clinical Information Engineering, Health Services Sciences, School of Public Health, Graduate School of Medicine, the University of Tokyo, Tokyo, Japan; Charles P. Darby Children’s Research Institute, UNITED STATES

## Abstract

**Background:**

Vitamin D has been reported to affect both innate, and acquired immunity with immune cells such as dendritic cells having the vitamin D receptors. The co-occurrence of the high prevalence of allergic diseases and vitamin D deficiency globally documented in recent decades, has prompted a hypothesis on whether there is a reasonable association between them.

**Objective:**

To investigate the association between serum vitamin D deficiency and allergic symptoms.

**Methods:**

Historical cohort. On a cohort study for the association between desert dust exposure and allergic symptoms in 3,327 pregnant women during spring and fall in 2011–2013 in Japan conducted as an adjunct study to the Japan Environment and Children’s Study, we promptly acquired subjects’ daily allergic symptom scores by sending a web-based questionnaire to each participant on some days. Of the 29,434 answers provided by 3,327 participating pregnant women, we extracted 13,356 answers from 1,475 pregnant women that were answered within a 3-month period after blood samplings. And we measured 25(OH)D levels on those samples to investigate the association between their vitamin D deficiency (serum 25(OH)D < 20ng/mL) and the occurrence of any allergic symptom (allergic symptom score> 0) within 3 months.

**Results:**

Serum 25(OH)D was less than 20ng/mL in 1,233 of 1,745 samples (70.7%). The adjusted odds ratio (aOR) for occurrence of any allergic symptom in deficient cases compared with non-deficient cases was 1.33 (95% CI: 1.07–1.64, p = 0.01). Further, vitamin D deficiency significantly enhanced the risk increase at desert dust events and at pollen exposure (p-values for interaction <0.1).

**Conclusion:**

We confirmed the association between serum vitamin D deficiency and allergic symptoms in Japanese pregnant women.

## Introduction

Allergic diseases are major public health problems worldwide with an increasing prevalence in the past 40 years[1,2], with their causes unknown. Many hypotheses link the allergy epidemic to stringent hygiene in early life, replacement of a traditional way of living with a westernized lifestyle, and a more accelerated pace of life. We have demonstrated that desert dust exposure is a risk factor for exacerbations of allergy symptoms[3]. However, some individuals were not affected at all by this extrinsic exposure, suggesting a partial role of the host, too. It would appear that these susceptible individuals, with intrinsic factors have increased in recent years.

As a consequence of indoor occupations followed by reduced exposure to sunlight, concerns have been raised that vitamin D deficiency is widespread in developed countries. Vitamin D has been reported to affect both innate, and acquired immunity through immune cells having the vitamin D receptors, such as dendritic cells and regulatory T cells[4].

In the present study, we hypothesized that vitamin D deficiency could be a cause of the excessive immune reactions at events such as desert dust exposure, and examined the association between serum vitamin D status and allergic symptom exacerbation, with respect to Asian dust events or pollen dispersion in pregnant women, by measuring frozen-stored serum 25(OH)D levels of pregnant women who were in a panel study where they responded to questionnaires about their allergic symptoms on the day repeatedly during spring and fall.

The study protocol was registered at UMIN000010826, and was published[5].

## Methods

### Study design

Historical cohort. The study was started in 2011 as the first part of an adjunct study of the Japan Environment and Children’s Study (JECS) to examine the effects of desert dust exposure on allergic symptoms in pregnant women, effect-modification potential of other pollutants and pollens, and to identify vulnerable populations for dust events in areas within Kyoto, Toyama, and Tottori, Japan[3,5–7]. The JECS participants from these regions who agreed to participate in the adjunct study were enrolled prior to delivery of their child. The details of the study design have been described elsewhere[3,5–7]. Briefly, questionnaires asking about allergic symptoms on the day were sent out during the Asian dust seasons (Feb-May and Oct-Nov) on Asian dust days and on several other randomly selected days for each participant during her pregnancy. The total number of questionnaires sent out was 41,020, of which 29,434 (71.8%) were completed within the set time limit of 28 hours after the questionnaire was sent out[3]. They also volunteered serum samples three times during pregnancy; once per pregnant trimester[7].

**Fig 1:**
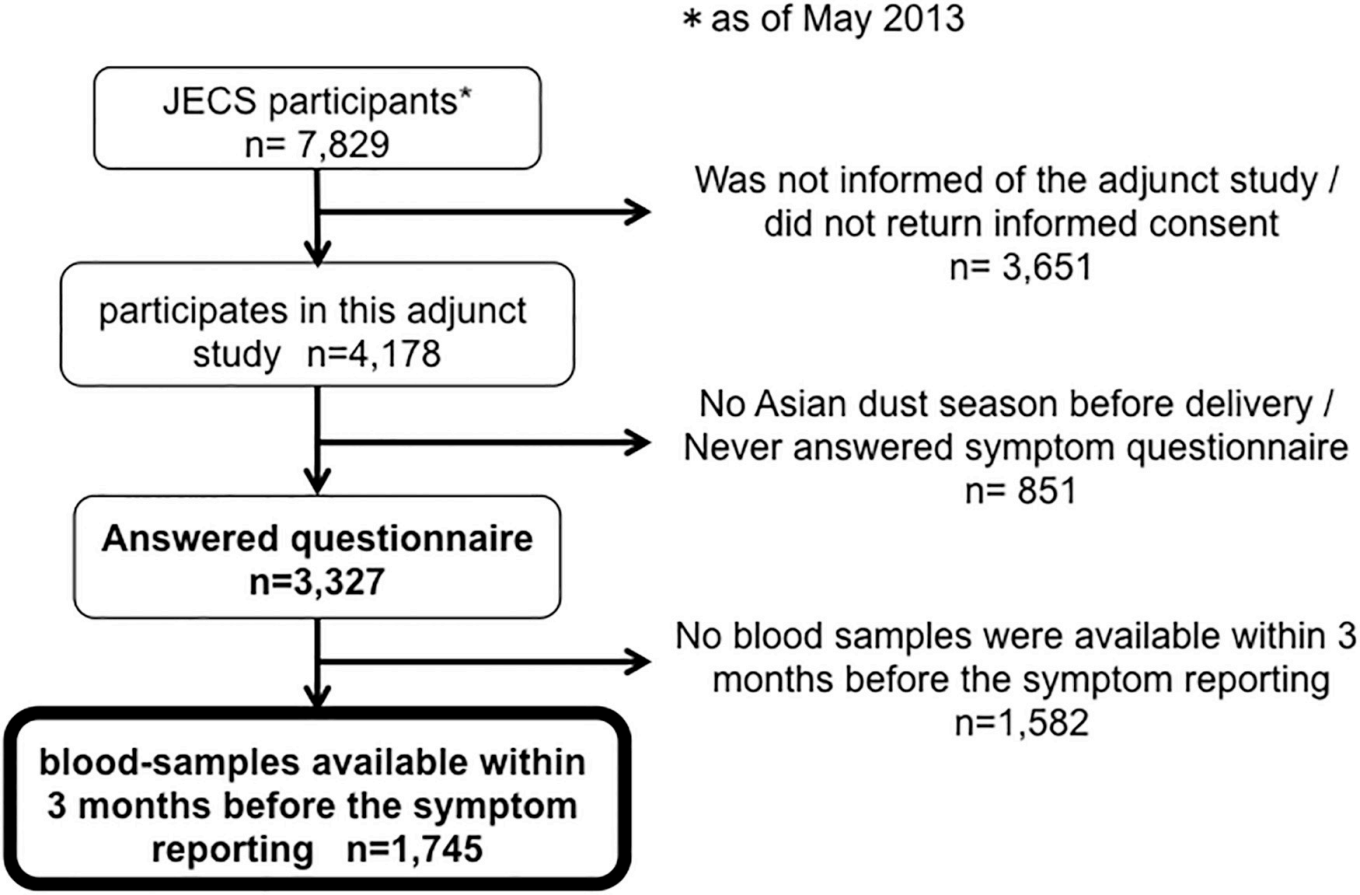
Extraction of subjects in the adjunct study of the Japan Environment and Children’s Study (JECS). Of 7,829 JECS participants as of May 2013 in Kyoto, Toyama, and Tottori, 4,178 participants presented informed consent to the adjunct study, and 3,327 participants responded to symptom questionnaire during the Asian dust season. Of them, we extracted responses of 1,745 participants that were answered within 3 months after their blood-sampling.

**Fig 2:**
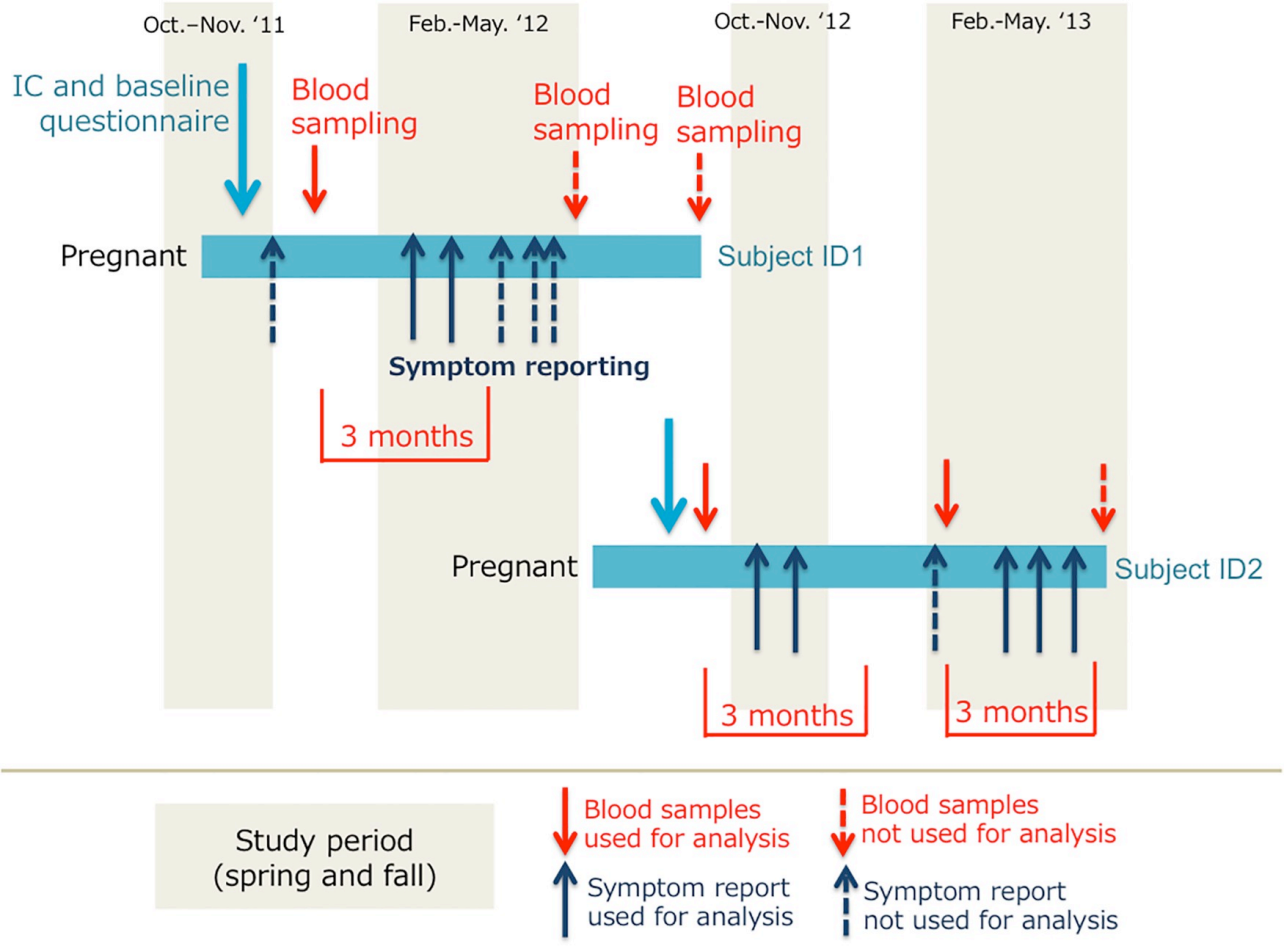
Study design. Questionnaires asking about allergic symptoms on the day were sent out during the Asian dust seasons (Feb-May and Oct-Nov) on Asian dust days and on several other randomly selected days for each participant during her pregnancy. Serum samples were drawn three times during pregnancy; once per pregnant trimester. Responses that were answered within 3 months after their blood-sampling were extracted for the analysis.

Of the 29,434 responses on 3,327 pregnant women, we extracted responses that were answered within 3 months after their blood-sampling (Figs 1 and 2). And we measured 25(OH)D levels in those samples to investigate the association between their vitamin D deficiency (serum 25(OH)D < 20ng/mL) and the occurrence of any allergic symptoms (allergic symptom score> 0). The study-protocol was approved by the ethics committees of Kyoto University, University of Toyama, and Tottori University.

### Measurements

#### Demographics

Upon enrolment, baseline information such as history of diagnosis of asthma or other allergic diseases was collected via a web-based questionnaire[5]. In addition, various lifestyle parameters, including diet, housing environment, socioeconomic background, and serum IgE to common allergens, were obtained typically during their 1^st^ trimesters[7]. The details of measured items are described elsewhere[3,5,7].

#### Vitamin D levels

Serum 25(OH)D was measured by a two-sandwich RIA method[8]. Biochemical analysis of blood was performed on serum samples at SRL laboratories. The serum samples were kept frozen (-30 °C) until analysis. 25(OH)D was measured using the 25-hydroxyvitamin D 125I RIA kit. The validated measurement range was 6–99900000 ng/ml.

#### Allergic symptoms

A self-administered questionnaire including items from the Japanese version of the Allergy Control Score [9,10] was sent to participants via mobile phone and/or personal computer. The Allergy Control Score consists of items pertaining to daily nasal, ocular, and lung symptoms, which are measured on a 4-point scale (absent, mild, moderate, and severe) [9,10].

#### Covariates

We examined the following factors including the model based on our previous report[3]: Asian dust, SO_2_, pollen count, lowest temperature on the day, temperature difference within the day, humidity, seasonality (spring or autumn), cedar-pollen-specific IgE class, and house-dust-mite (Dermatophagoides pteronyssinus)-specific IgE class. Details are shown elsewhere[3].

### Statistical analysis

Although there is little agreement on the definition of vitamin D insufficiency especially for non-musculoskeletal health, the Institute of Medicine panel concluded that circulating levels of 25(OH)D >20 ng/mL are sufficient with respect to skeletal health[11]. *A priori* we defined vitamin D deficiency as serum 25(OH)D less than 20 ng/mL of serum 25(OH)D in the protocol based on previous studies conducted on the Japanese people[12,13]. For occurrences of any symptoms (symptom score exceeding 0), we estimated an odds ratio for an effect measure that compares the symptom occurrence between vitamin D deficient cases (25(OH)D <20 ng/mL) and non-deficient cases, using the Generalized Estimating Equations (GEE) logistic regression analysis. We then included the interaction term of desert dust exposure and vitamin D deficiency or pollen exposure and vitamin D deficiency to investigate if the vitamin D sufficiency changes the risk of Asian dust or pollen exposure for the occurrence of allergic symptoms.

The robustness of the main results was tested by performing the following sensitivity analyses. First, we tried a cut-off value of 30 ng/mL serum 25(OH)D as vitamin D deficiency, and we also tried treating 25(OH)D as a continuous variable. Then we treated the outcome as a continuous variable (log-transformed symptom scores) using a linear mixed model.

All analysis were performed using SAS software, version 9.3 (SAS Institute), and 2-sided P < 0.05 was considered statistically significant (P <0.1 for interaction terms).

## Results

### Subjects

Of the 29,434 answers on 3,327 pregnant women who participated in the study, we extracted 13,356 answers for 1,475 pregnant women, that were responded within 3 months after their blood-sample (Fig 1). Table 1 summarize the characteristics of extracted subjects. All were pregnant, in a wide age range from teens to over 45 years, and from various socioeconomic backgrounds. Approximately half exhibited positive IgE to cedar pollen and nearly half to dust mite allergen. Participant characteristics were similar to those reported by the Japanese government in 2012, except that the proportion of current smokers was lower among the study participants (1.6–2.7%) compared with those in the government report (12.8% for women in their 20s and 16.6% for women in their 30s) [14,15], and that the proportion of participants with an education level up to junior high school completion was lower (1.6–3.3%) compared to that in the government report (6.0%)[16,17]. Overall, we believe the extracted cohort was considered a good representation of pregnant women in Japan.

**Table 1:** Characteristics of subjects.

	Kyoto n = 464	Toyama n = 744	Tottori n = 267
**Age (years)** ^a^ ^b^ ^**n = 1457 459,734,264**^	32.2±4.5	31.0±4.8	31.0±4.5
**Height (cm)** ^a^ ^b^ ^**n = 455, 718, 257**^	159.0±5.5	158.7±5.2	157.6±5.0
**BMI before pregnancy** ^a^ ^b^ ^**n = 453, 734, 262**^	20.8±2.9	21.0±2.8	20.7±2.7
**History of allergic diseases**	n (%)	n (%)	n (%)
Asthma	52 (11.2)	81 (10.9)	39 (14.6)
Allergic rhinitis	254 (45.3)	463 (37.8)	157 (41.2)
Atopic dermatitis	87 (18.8)	125 (16.8)	46(17.2)
**Serum positive IgE** ^c^	n (%)	n (%)	n (%)
Japanese Cedar Pollen positive	283 (61.0)	397 (53.4)	126 (47.2)
House Dust Mite positive	233 (50.2)	373 (50.1)	118 (44.2)
Sample not available	15 (3.2)	23 (3.1)	13 (4.9)
**Smoking history** ^b^	n (%)	n (%)	n (%)
Never smoked	297 (64.0)	432 (58.1)	163 (61.0)
Stopped before pregnancy	108 (23.3)	195 (26.2)	63 (23.6)
Stopped after pregnancy	34 (7.3)	69 (9.3)	24 (9.0)
Current smoker	7 (1.4)	18 (2.5)	6 (1.5)
Blank ^d^	18 (3.9)	30 (4.0)	11 (4.1)
**Partner’s smoking history** ^b^	n (%)	n (%)	n (%)
Never smoked	163 (35.1)	209 (28.1)	69 (25.8)
Stopped before pregnancy	129 (27.8)	183 (24.6)	68 (25.5)
Stopped after pregnancy	9 (1.9)	14 (1.9)	3 (1.1)
Current smoker	140 (31.5)	302 (40.8)	112 (45.0)
Blank ^d^	23 (5.0)	36 (4.8)	15 (5.6)
**Education level** ^b^	n (%)	n (%)	n (%)
Junior high school	7 (1.5)	24 (3.2)	8 (3.0)
High school	84 (18.1)	172 (23.1)	79 (29.6)
Vocational school	65 (14.0)	167 (22.4)	61 (22.8)
College (2 years)	110 (23.7)	128 (17.2)	43 (16.1)
High vocational School	2 (1.1)	4 (2.7)	7 (3.0)
College (4 years)	165 (35.6)	214 (28.8)	57 (21.3)
Graduate school	26 (5.6)	15 (2.0)	4 (1.5)
Blank ^d^	5 (1.1)	20 (2.7)	8 (3.0)
**Family income /year** ^b^	n (%)	n (%)	n (%)
Under $20,000	11 (2.4)	11 (1.5)	15 (5.6)
$20,000–40,000	125 (26.9)	209 (28.1)	99 (37.1)
$40,000–60,000	157 (33.8)	261 (35.1)	67 (25.1)
$60,000–80,000	85 (18.3)	117 (15.7)	41 (15.4)
$80,000–100,000	39 (8.4)	62 (8.3)	12 (4.5)
Above $100,000	27 (5.8)	26 (3.5)	13 (4.9)
Blank ^d^	20 (4.3)	58 (7.8)	20 (7.5)
**Frequency exposed to sunlight**	n (%)	n (%)	n (%)
Rarely	43 (9.3)	161 (22.0)	51 (19.1)
1 to 2 days a week	94 (20.3)	192 (25.8)	68 (25.5)
3 to 4 days a week	117 (25.2)	165 (22.2)	55 (20.6)
More than 5 days a week	172 (37.1)	163 (21.9)	63 (23.6)
Blank ^d^	38 (8.2)	60 (8.1)	30 (11.2)
UV block usage	n (%)	n (%)	n (%)
Never expose bare skin to sunlight	10 (2.2)	27 (3.6)	5 (1.9)
Sometimes use UV-blocks	275 (59.3)	362 (48.7)	123 (46.1)
Rarely use UV-blocks	120 (25.9)	262 (35.2)	104 (39.0)
Blank ^d^	59 (12.7)	93 (12.5)	35 (13.1)

^a^ Mean ± SD

^b^ referred to JECS (tentative data)

^c^ referred to JECS (fixed data)

^d^ Blank includes those who did not return the answer sheet.

### Vitamin D sufficiency status

**Fig 3:**
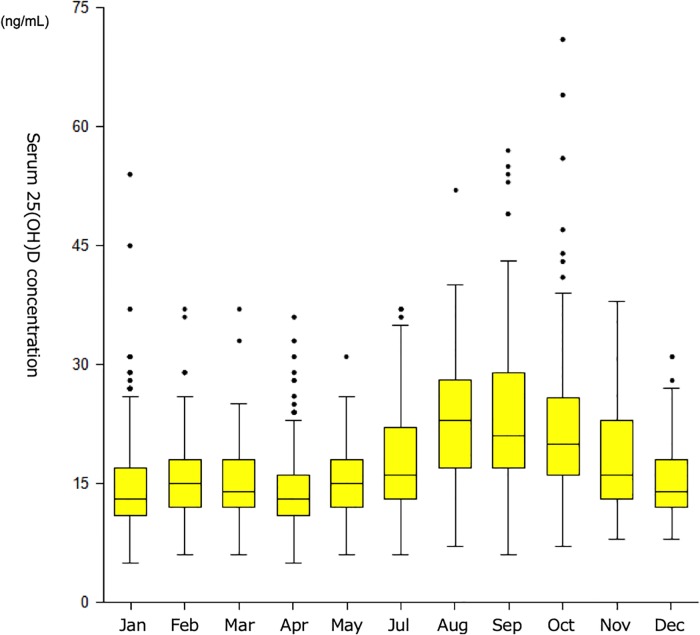
Serum 25(OH)D levels in relation to sampled months. Serum 25(OH)D was less than 20ng/mL in 1,233 of 1,745 samples (70.7%). There was a clear seasonal change with a peak at the end of summer and a trough in early spring. The median level of serum 25(OH)D in each season was 15, 14, 19, and 20 ng/mL in winter (Dec-Feb), spring (Mar-May), summer (Jul-Aug) and autumn (Sep-Nov).

Serum 25(OH)D was less than 20ng/mL in 1,233 of 1,745 samples (70.7%), and 8.0% (139 of 1,745 samples) showed even less than 10 ng/mL which is characterized as severe vitamin D deficiency. Consistent with previous reports, there was a clear seasonal change with a peak at the end of summer and a trough in early spring[18] (Fig 3), both in allergic and non-allergic pregnant women (S1 and S2 Figs). The median level of serum 25(OH)D in each season was 15, 14, 19, and 20 ng/mL in winter (Dec-Feb), spring (Mar-May), summer (Jul-Aug) and autumn (Sep-Nov), respectively.

### Association between vitamin D deficiency and allergic symptoms

GEE logistic regression analysis with an exchangeable correlation matrix showed that pregnant women were more likely to develop allergic symptoms (symptom score >0) when they were vitamin D deficient (i.e. 25(OH)D < 20ng/mL) compared with non-deficient subjects (odds ratio [OR] 1.29; 95% confidence interval [95%CI]: 1.08–1.54, p = 0.005, adjusted OR 1.33 (95% CI: 1.07–1.64, p = 0.009), Table 2). The increased odds of the vitamin D deficiency was observed independently of the pregnancy trimesters, that is, including the trimester in the adjusted model did not change the estimated OR (adjusted OR for allergic symptom development of vitamin D deficiency: 1.29 (95%CI: 1.05–1.59, p = 0.0167); and the 25(OH)D deficiency was a consistent risk factor for the allergic symptoms through the trimesters (S1 and S2 Tables). Sensitivity analysis showed that this was true with a cut-off value of 30 ng/mL serum 25(OH)D, which is often described as vitamin D insufficiency level, and the OR for symptom development decreased as the vitamin D level increased (OR 0.80; 95%CI: 0.64–1.00, p = 0.053 for those with 20–30 ng/mL, and OR 0.68; 95%CI: 0.47–0.99, p = 0.043 for those with <30 ng/mL, S3 Table).

**Table 2:** Odds ratio (OR) and its 95% Confidence Interval (95%CI) for allergic symptom development.

	OR	95% CI			P value
Vitamin D deficiency ^a^	1.33	1.07	–	1.64	.009
IgE to cedar pollen (per class increase)	1.28	1.21	–	1.35	< .001
IgE to house dust mite (per class increase)	1.10	1.03	–	1.18	.008
Age (compared with >40 years)					
<20 years	0.81	0.19	–	3.55	.781
20–25 years	1.82	1.01	–	3.27	.046
25–30 years	1.49	0.95	–	2.35	.085
30–35 years	1.62	1.04	–	2.53	.034
35–40 years	1.47	0.93	–	2.32	.103
BMI before pregnancy (compared with >25)					
<18	1.00	0.68	–	1.47	.995
18–25	1.14	0.82	–	1.60	.436
Family income (per 20,000 increase)	0.95	0.87	–	1.01	.113
Smoking status of subjects (Compared with current smoker)					
Never smoker	0.98	0.54	–	1.79	.957
Stopped before pregnancy	1.12	0.61	–	2.07	.708
Stopped after pregnancy	1.04	0.54	–	1.97	.915
Smoking status of subjects’ partners (Compared with current smoker)					
Never smoker	1.01	0.81	–	1.26	.940
Stopped before pregnancy	1.01	0.81	–	1.25	.956
Stopped after pregnancy	0.51	0.29	–	0.90	.020
Location (Compared with Kyoto)					
Toyama	0.99	0.81	–	1.21	.920
Tottori	1.03	0.80	–	1.34	.795

Adjusted by desert dust, pollen counts, SO_2_, humidity, lowest temperature of the day, temperature difference within the day, and month on the day.

Vitamin D deficiency was defined as serum 25(OH)D level < 20 ng/mL on serum samples taken within 3 months before the symptom response.

IgE class to cedar pollen and IgE class to house dust mite were treated as ordered categorical. The OR per one class increase for each is shown.

Age was categorized into 6 groups by 5 years and was treated as categorical.

BMI before pregnancy was categorized into 3 groups, and was treated as categorical.

Family income was categorized into 6 groups and was treated as ordered categorical.

### Vitamin D deficiency as an allergic-symptom risk modifier at events

The risk increases during Asian dust events appeared more evident in vitamin D deficient subjects compared with vitamin D non-deficient subjects (OR 1.15, 95%CI: 1.04–1.27 for deficient subjects, and OR 1.10, 95%CI: 0.88–1.36 for non-deficient subjects), and so did the one by pollen exposure (OR 1.45, 95%CI: 1.29–1.63 for deficient subjects, and OR 1.18, 95%CI: 0.90–1.54 for non-deficient subjects) (S4 and S5 Tables). The models with interaction terms of ‘dust level’ and ‘serum 25(OH)D deficiency’ or ‘pollen level’ and ‘serum 25(OH)D deficiency’ showed statistically significant (p < 0.1) interaction (Tables 3 and 4).

**Table 3:** Odds ratio (OR) and its 95% Confidence Interval (95%CI) for allergic symptom development with the interaction term of vitamin D deficiency and desert dust.

	OR	95% CI			P value
Vitamin D deficiency ^a^	1.03	0.97	–	1.09	.288
Desert dust ^b^	1.04	1.01	–	1.06	.003
Interaction term	1.03	0.99	–	1.07	.098

Those who were seropositive for IgE to JCP and went outdoors on the day were included in the analysis.

Adjusted by pollen counts (log-transformed and standardized), humidity, lowest temperature of the day, temperature difference within the day, IgE to house dust mite, domestic income, smoking status, age, and BMI before pregnancy.

IgE class to house dust mite was treated as ordered categorical.

Age was categorized into 6 groups by 5 years, and was treated as categorical.

BMI before pregnancy was categorized into 3 groups, and was treated as categorical.

Family income was categorized into 6 groups and was treated as ordered categorical.

^a^ Vitamin D deficiency was defined as serum 25(OH)D level < 20 ng/mL on serum samples taken within 3 months before the symptom response.

^b^ Desert dust level was log-transformed and standardized.

**Table 4:** Odds ratio (OR) and its 95% Confidence Interval (95%CI) for allergic symptom development with the interaction term between vitamin D deficiency and pollen exposure.

	OR	95% CI			P value
Vitamin D deficiency ^a^	1.05	0.99	–	1.11	.125
Pollen ^b^	1.09	1.06	–	1.12	< .001
Interaction term	1.05	1.00	–	1.11	.059

Those who were seropositive for IgE to JCP and went outdoors on the day were included in the analysis.

Adjusted by desert dust (log-transformed and standardized), humidity, lowest temperature of the day, temperature difference within the day, IgE to house dust mite, family income, smoking status, age, and BMI before pregnancy.

IgE class to house dust mite was treated as ordered categorical.

Age was categorized into 6 groups by 5 years, and was treated as categorical.

BMI before pregnancy was categorized into 3 groups, and was treated as categorical.

Family income was categorized into 6 groups and was treated as ordered categorical.

^a^ Vitamin D deficiency was defined as serum 25(OH)D level < 20 ng/mL on serum samples taken within 3 months before the symptom response.

^b^ Pollen count was log-transformed and standardized.

## Discussion

In this study, we showed that pregnant women are significantly more likely to have self-reported allergic symptoms when they have deficient level of 25(OH)D (< 20ng/mL) compared with non-deficient women. The increased risks of allergic symptoms during Asian dust exposure and pollen exposure were more evident in vitamin D deficient subjects compared with vitamin D sufficient subjects. Our finding supports the hypothesis that the recent epidemic of allergy could partly be caused by vitamin D deficiency, suggesting that we may be able to reduce the symptoms by approximately 30% by ensuring a level of 20 ng/mL of serum 25(OH)D level for pregnant women. The impact would be large because vitamin D deficiency is observed wide-spread; Serum 25(OH)D was less than 20ng/mL in 1,233 of 1,745 samples (70.7%) in our study, and this was consistent with reports of pregnant women from Japan and other Asian countries[19,20].

Some epidemiologic studies suggest a protective effect of higher vitamin D exposure against allergic diseases, while other results point to increased risk or no associations with vitamin D exposure. The latest systematic review for vitamin D supplementation for pregnant women, breastfeeding women, and infants concluded that the supplementation did not decrease the risk of developing allergic diseases[21]. On the other hand, another latest systemic review revealed that vitamin D supplementation reduced the frequency of asthma exacerbations[22]. Besides of the heterogeneity in the definition of outcomes and timings of vitamin D measurements, there may be other potential reasons for the controversial findings. Hawrylowicz *et al*. recently showed an interesting hypothesis that the apparent beneficial association of vitamin D in allergies *in vivo*, which in many patients is a T_H_2-type cytokine pathology, suggests that the direct action of vitamin D in promoting T_H_2 lymphocyte responses is less important *in vivo* than other vitamin D–mediated mechanisms (eg, vitamin D upregulated production of soluble decoy receptor, an inhibitor of IL-33 *in situ* in the airways mucosa, and the induction of regulatory mechanisms) in inhibiting T_H_2-type cytokine responses[23]. Vitamin D supplementations may not reduce the risk of developing allergic diseases[21], but may reduce the inflammation (exacerbations of symptoms) in people with allergies. Our results that Asian dust, which includes silica and other possible danger signals[3] that may cause IL-33 release, exhibits adjuvant-like effects in pollen-sensitized pregnant women[3], and this was evident in vitamin D deficient women supports this hypothesis. Further work needs to be done in this field to elucidate the mechanisms.

The strengths of this study include a relatively large dataset on a population-based cohort resulting in high generalizability to pregnant women in Japan. Other strengths include the fact that the lists of symptoms were collected repeatedly during Asian dust/pollen season while their serum IgE levels were also measured. This enabled us to perform detailed analysis for symptom developments at events. And by sending a web-based questionnaire on the day, which required a response within a set time limit using a mobile phone or personal computer, we ensured a high response rate (72%) and reduced recall misclassifications of symptoms.

This study has three major limitations. The most notable is our inability to exclude the possibility of reverse causality for the association between allergic symptom and vitamin D deficiency, as is often the case with observational studies; that is, the pregnant women who had severe symptoms might not have dared to go outdoors to avoid pollen exposure, and this might have resulted in low 25(OH)D levels in the serum due to reduced sunlight exposure. Second, allergic symptoms were self-reported and not confirmed by medical professionals. In other words, symptoms were not distinguished by origin (i.e., allergy, infection, or other). Thus, our speculation regarding allergic symptoms is based on timing of the onset of symptoms (i.e., due to pollen or Asian dust) and vulnerability of participants (i.e., positive serum IgE to pollen). The third limitation relates to generalizability. We investigated pregnant women, whose immune responses as well as vitamin D status might have been altered due to pregnancy. Further studies should be performed to verify whether our findings also apply to the non-pregnant population.

In conclusion, pregnant women were significantly more likely to have allergic symptoms if their 25(OH)D level was < 20ng/mL, compared with non-deficient subjects (OR 1.28 (1.04–1.56)). We may be able to reduce the symptoms by approximately 30% by ensuring a level of 20 ng/mL of serum 25(OH)D level for pregnant women. The risk increase during Asian dust events or pollen exposure was more evident in vitamin D deficient pregnant women compared with vitamin D non-deficient pregnant women. This adds to the growing evidence of the association between vitamin D insufficiency and allergic diseases.

## Supporting information

S1 Fig Serum 25(OH)D levels in relation to sampled months on subjects with/without history of allergic rhinitis. There was a clear seasonal change with a peak at the end of summer and a trough in early spring regardless of past history of allergic rhinitis.
(TIFF)

S2 Fig Serum 25(OH)D levels in relation to sampled months on subjects seropositive/seronegative to Japanese Cedar Pollen (JCP). There was a clear seasonal change with a peak at the end of summer and a trough in early spring regardless of the presence of serum IgE to JCP.
(TIFF)

S1 Table Odds ratio (OR) and its 95% Confidence Interval (95%CI) for allergic symptom development of vitamin D deficiency in their 1^st^ trimester. (DOCX)

S2 Table Odds ratio (OR) and its 95% Confidence Interval (95%CI) for allergic symptom development of vitamin D deficiency in their 2^nd^ trimester. (DOCX)

S3 Table Odds ratio (OR) and its 95% Confidence Interval (95%CI) for allergic symptom development on cases with sufficient in vitamin D (> = 30 ng/mL) and on cases with insufficient vitamin D (20–30 ng/mL) compared with cases deficient in vitamin D (< 20 ng/mL). (DOCX)

S4 Table Odds ratio (OR) and its 95% Confidence Interval (95%CI) for allergic symptom development compared with cases deficient in vitamin D (< 20 ng/mL). (DOCX)

S5 Table Odds ratio (OR) and its 95% Confidence Interval (95%CI) for allergic symptom development in Vitamin D non-deficient subjects (25(OH)D > = 20 ng/mL). (DOCX)
