# Supplementary figures and images for: Association between vitamin D deficiency and allergic symptom in pregnant women
Source: PLoS One. 2019 Apr 10;14(4):e0214797. doi: 10.1371/journal.pone.0214797 (PMC6457537; doi:10.1371/journal.pone.0214797)

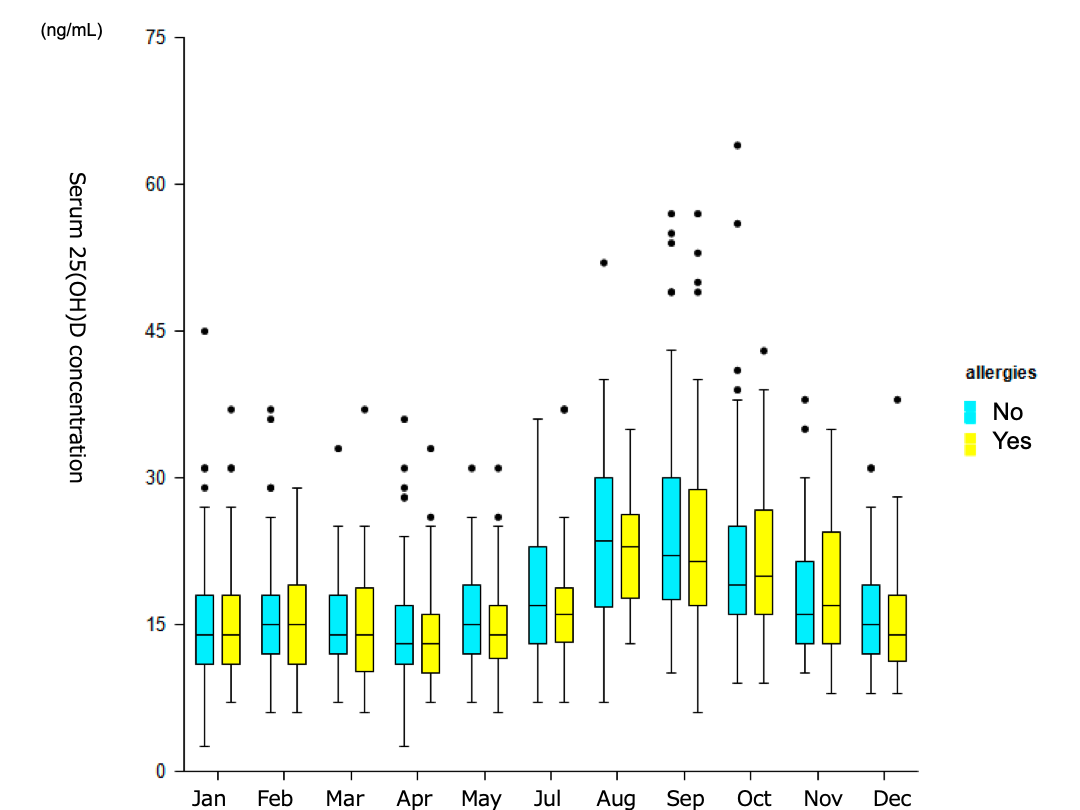

Supplement: S1 Fig — There was a clear seasonal change with a peak at the end of summer and a trough in early spring regardless of past history of allergic rhinitis. (TIFF) [file pone.0214797.s001.tiff]

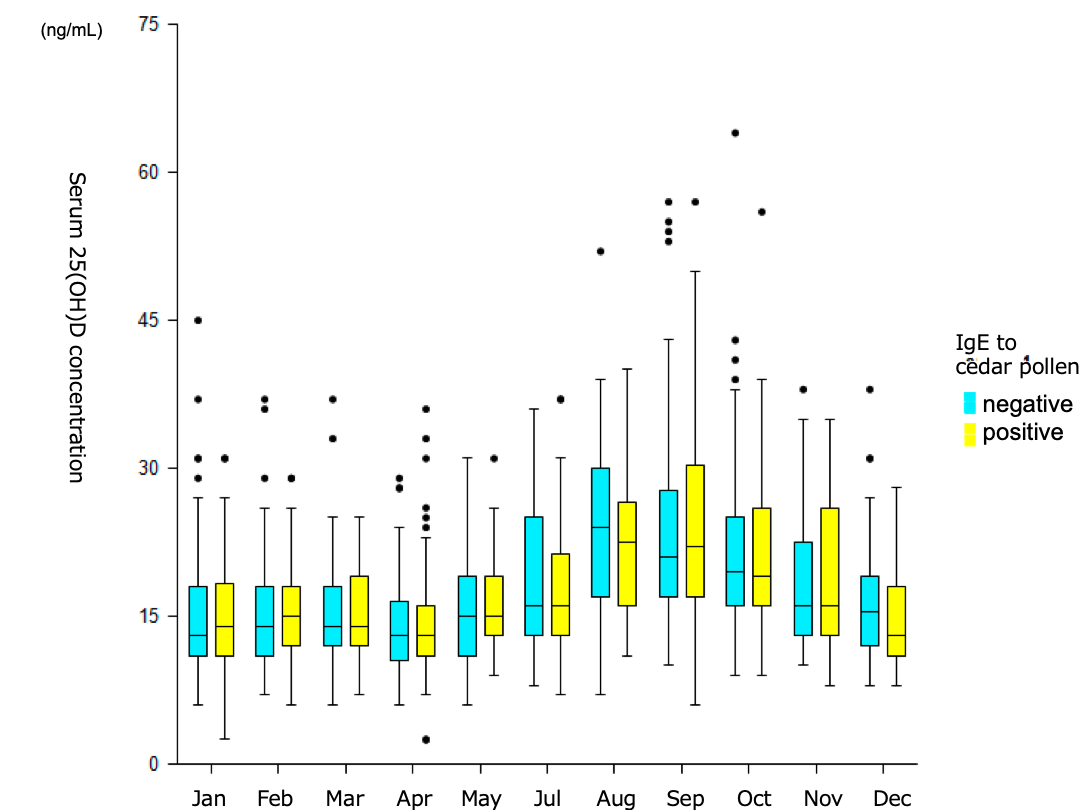

Supplement: S2 Fig — There was a clear seasonal change with a peak at the end of summer and a trough in early spring regardless of the presence of serum IgE to JCP. (TIFF) [file pone.0214797.s002.tiff]
