# Supplementary material for: Association between vitamin D deficiency and allergic symptom in pregnant women
Source: PLoS One. 2019 Apr 10;14(4):e0214797. doi: 10.1371/journal.pone.0214797 (PMC6457537; doi:10.1371/journal.pone.0214797)
Supplement: S1 Table — (DOCX) [file pone.0214797.s003.docx]

**S1 Table. Odds ratio (OR) and its 95% Confidence Interval (95%CI) for allergic symptom development of vitamin D deficiency in their 1^st^ trimester.**

|  | OR | 95% CI | | | P value |
| --- | --- | --- | --- | --- | --- |
| Vitamin D deficiency (< 20 ng/mL) | 1.26 | 0.88 | – | 1.80 | .209 |
| IgE to cedar pollen (per class increase) | 1.37 | 1.24 | – | 1.50 | <.001 |
| IgE to house dust mite (per class increase) | 1.17 | 1.03 | – | 1.32 | .019 |
| Age (compared with >40 years) |  |  |  |  |  |
| <20 years | 0.12 | 0.05 | – | 0.28 | <.001 |
| 20-25 years | 1.48 | 0.50 | – | 4.39 | .478 |
| 25-30 years | 1.64 | 0.74 | – | 3.67 | .226 |
| 30-35 years | 1.61 | 0.72 | – | 3.59 | .248 |
| 35-40 years | 1.39 | 0.61 | – | 3.17 | .432 |
| BMI before pregnancy  (compared with >25) |  |  |  |  |  |
| <18 | 0.71 | 0.36 | – | 1.38 | .313 |
| 18-25 | 1.13 | 0.63 | – | 2.04 | .681 |
| Family income (per 20,000 increase) | 1.00 | 0.87 | – | 1.15 | .976 |
| Smoking status of subjects  (Compared with current smoker) |  |  |  |  |  |
| Never smoker | 0.78 | 0.36 | – | 1.67 | .522 |
| Stopped before pregnancy | 1.30 | 0.58 | – | 2.93 | .526 |
| Stopped after pregnancy | 1.16 | 0.50 | – | 2.71 | .725 |
| Smoking status of subjects’ partners  (Compared with current smoker) |  |  |  |  |  |
| Never smoker | 1.17 | 0.79 | – | 1.73 | .425 |
| Stopped before pregnancy | 0.99 | 0.66 | – | 1.48 | .967 |
| Stopped after pregnancy | 0.37 | 0.19 | – | 0.74 | .005 |

Vitamin D deficiency was defined as serum 25(OH)D level < 20 ng/mL on serum samples taken within 3 months before the symptom response.

Adjusted by season, Asian dust event, pollen dispersal, humidity, lowest temperature of the day, temperature difference within the day, and humidity, month on the day.

Asian dust was defined as > 0.07/km. The cut-off value was based on the value used in the previous report[3].

Pollen dispersal was defined as >40/cm^2^. The cut-off value was based on the value used in the previous report[3].

IgE class to cedar pollen and IgE class to house dust mite were treated as ordered categorical. The OR per one class increase for each is shown.

Age was categorized into 6 groups by 5 years and was treated as categorical.

BMI before pregnancy was categorized into 3 groups, and was treated as categorical.

Family income was categorized into 6 groups and was treated as ordered categorical.
