# Supplementary material for: Association between vitamin D deficiency and allergic symptom in pregnant women
Source: PLoS One. 2019 Apr 10;14(4):e0214797. doi: 10.1371/journal.pone.0214797 (PMC6457537; doi:10.1371/journal.pone.0214797)
Supplement: S3 Table — (DOCX) [file pone.0214797.s005.docx]

**S3 Table. Odds ratio (OR) and its 95% Confidence Interval (95%CI) for allergic symptom development on cases with sufficient in vitamin D (>=30 ng/mL) and on cases with insufficient vitamin D (20-30 ng/mL) compared with subjects deficient in vitamin D (< 20 ng/mL)**

|  | OR | 95% CI | | | P value |
| --- | --- | --- | --- | --- | --- |
| Vitamin D status |  |  |  |  |  |
| Insufficiency (20-30 ng/mL) | 0.80 | 0.64 | - | 1.00 | .053 |
| Sufficiency (>30 ng/mL) | 0.68 | 0.47 | - | 0.99 | .043 |

Adjusted by BMI, age, IgE to cedar pollen, IgE to dust-mite, family income, smoking status of the subject, smoking status of partners, desert dust, pollen counts, SO_2_, humidity, lowest temperature of the day, temperature difference within the day, location (Kyoto, Toyama, or Tottori), and month on the day.

IgE class to cedar pollen and IgE class to house dust mite were treated as ordered categorical. The OR per one class increase for each is shown.

Age was categorized into 6 groups by 5 years and was treated as categorical.

BMI before pregnancy was categorized into 3 groups, and was treated as categorical.

Family income was categorized into 6 groups and was treated as ordered categorical.
